# Brain corticospinal tract abnormalities in aquaporin-4 seropositive neuromyelitis optica spectrum disorder with longitudinally extensive transverse myelitis

**DOI:** 10.1093/braincomms/fcaf257

**Published:** 2025-06-27

**Authors:** Chengyi Zhang, Yajun Yao, Jiali Sun, Yun Xu, Huabing Wang, Zhi-Zheng Zhuo, Yunyun Duan, Xinghu Zhang, Yaou Liu, Wencai Ding, De-Cai Tian

**Affiliations:** Department of Neurology, Beijing Tiantan Hospital, Capital Medical University, Beijing 100070, China; Department of Neurology, Beijing Anzhen Hospital, Capital Medical University, Beijing 100013, China; Department of Neurology, Beijing Tiantan Hospital, Capital Medical University, Beijing 100070, China; Department of Neurology, Beijing Tiantan Hospital, Capital Medical University, Beijing 100070, China; Department of Neurology, Beijing Tiantan Hospital, Capital Medical University, Beijing 100070, China; Department of Neurology, Beijing Tiantan Hospital, Capital Medical University, Beijing 100070, China; Department of Radiology, Beijing Tiantan Hospital, Capital Medical University, Beijing 100070, China; Department of Radiology, Beijing Tiantan Hospital, Capital Medical University, Beijing 100070, China; Department of Neurology, Beijing Tiantan Hospital, Capital Medical University, Beijing 100070, China; Department of Radiology, Beijing Tiantan Hospital, Capital Medical University, Beijing 100070, China; Department of Neurology, The Second Affiliated Hospital of Wannan Medical College, Wuhu 214002, China; Department of Neurology, Beijing Tiantan Hospital, Capital Medical University, Beijing 100070, China

**Keywords:** neuromyelitis optica spectrum disorder, corticospinal tract, aquaporin-4, longitudinally extensive transverse myelitis, fixel-Based analysis

## Abstract

Widespread white matter changes, including those in the corticospinal tract (CST), have been observed in patients with neuromyelitis optica spectrum disorder (NMOSD). However, whether these alterations originate within the brain or result from spinal cord damage remains unclear. To investigate the CST alteration in AQP4-IgG positive NMOSD with longitudinally extensive transverse myelitis (LETM), and evaluate its relevance to LETM via fixel-based analysis. AQP4+ LETM patients and healthy controls were from ongoing clinical and imaging patterns of neuroinflammation diseases in China cohort between December 2018 and September 2023. Fibre density, fibre cross-section, and the combined measure of fibre density and fibre cross-section were calculated in CST. Serum neurofilament light chain and glial fibrillary acidic protein levels were determined using single-molecule arrays. Multivariate linear model was used to assess associations in AQP4+ LETM between fixel parameters and clinical variables. 44 AQP4+ LETM and 48 healthy controls were included in the study. Among AQP4+ LETM, CST exhibited a decreased mean fibre cross-section (*P* = 0.001) and the combined measure of fibre density and cross-section (*P* = 0.004) compared to controls, particularly pronounced in the inferior segments. Negative associations were revealed: pyramidal function score with fibre density (*r* = −0.335, *P* = 0.037); LETM segments with fibre density (*r* = −0.429, *P* = 0.006); and the combined measure of fibre density and cross-section with duration of pyramidal tract impairment (*r* = −0.349, *P* = 0.032) and LETM segment (*r* = −0.384, *P* = 0.014). Serum neurofilament light chain and glial fibrillary acidic protein were elevated in AQP4+ LETM than healthy controls (*P* < 0.0001). Serum neurofilament light chain was negatively related to the combined measure of fibre density and cross-section (*r* = −0.428, *P* = 0.01) and fibre cross-section (*r* = −0.379, *P* = 0.025). Distinct reduction of fibre cross-section and the combined measure of fibre density and cross-section in brain CST were observed in AQP4+ LETM. The fixel parameters of CST in AQP4+ LETM were negatively correlated with the severity and duration of LETM impairment.

## Teaser

Zhang *et al*. report that patients with aquaporin-4 antibody-positive neuromyelitis optica spectrum disorder and longitudinally extensive transverse myelitis exhibit corticospinal tract atrophy. Fixel-based analysis reveals significant correlations between corticospinal degeneration and disease severity, duration and neurofilament light chain levels, highlighting neurodegenerative processes in neuromyelitis optica spectrum disorder.

## Graphical Abstract

**Graphical Abstract:**
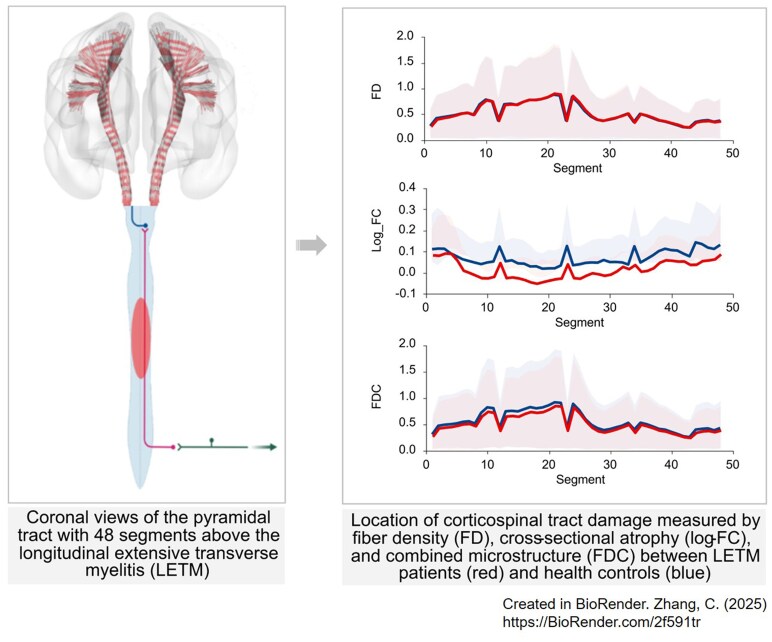

## Introduction

Aquaporin 4 (AQP4)-IgG-positive neuromyelitis optica spectrum disorder (AQP4 + NMOSD) is an autoimmune astrocytopathic disease that typically manifests as relapsing episodes of severe optic neuritis and myelitis.^1^ Longitudinally extensive transverse myelitis (LETM), defined on magnetic resonance imaging (MRI) as a lesion extending three or more vertebral segments, is the a highly characteristic radiological finding of AQP4+ NMOSD that usually causes severe physical disability.^2^ In AQP4+ NMOSD, spinal cord lesions had a median length of 6–10 vertebral segments, reaching a maximum of 17 segments.^3-5^

Spinal cord damage can initiate retrograde degeneration, leading to structural changes in the brain, particularly affecting long axonal tracts like the corticospinal tract (CST), which are susceptible to neurodegeneration following myelitis.^6,7^ Corticospinal neurons are the principal output of the sensorimotor cortex, responsible for relaying motor command signals to the spinal cord.^8^ Patients with spinal cord injury exhibit spatially and temporally varying changes in brain structure, including the CST.^9^ Moreover, reorganization of CST after spinal cord injuries were also found related with longer-term outcomes.^10^

Widespread cerebral white matter abnormalities, including the CST, have also been reported in NMOSD. CST damage in NMOSD was more severe compared to multiple sclerosis (MS) and exhibited spatial heterogeneity.^11-14^ It is unclear whether the damage to the CST in NMOSD originates in the brain or from spinal cord. Additionally, those with LETM in AQP4+ NMOSD (AQP4+ LETM) exhibit reduced brain volume with an accelerated rate of brain atrophy,^15,16^ which indicate the impact of LETM on the brain structural changes.

Currently, diffusion-weighted magnetic resonance imaging (dMRI) is one of the most reliable methods for assessing fibre tract integrity.^17^ Fixel-based analysis (FBA) stands out for its effectiveness in mitigating fibre cross effects and minimizing contamination from extra-axonal signals, distinguishing it from alternative dMRI analysis methods.^18^

Based on the Clinical and Imaging Patterns of Neuroinflammation Diseases in China (CLUE) cohort, the aim of this study is to explore the relationship between CST degeneration and spinal cord damage in NMOSD patients using FBA and evaluate its correlation with clinical metrics and blood biomarkers. By quantifying fibre density and fibre cross-section changes in CST, we assess whether these alterations are related to pyramidal tract dysfunction and neurodegeneration triggered by spinal cord injury.

## Materials and methods

The study received approval from the Beijing Tiantan hospital’s ethics committee, and informed consent was obtained from all patients during data collection.

### Study patients

**Figure 1:**
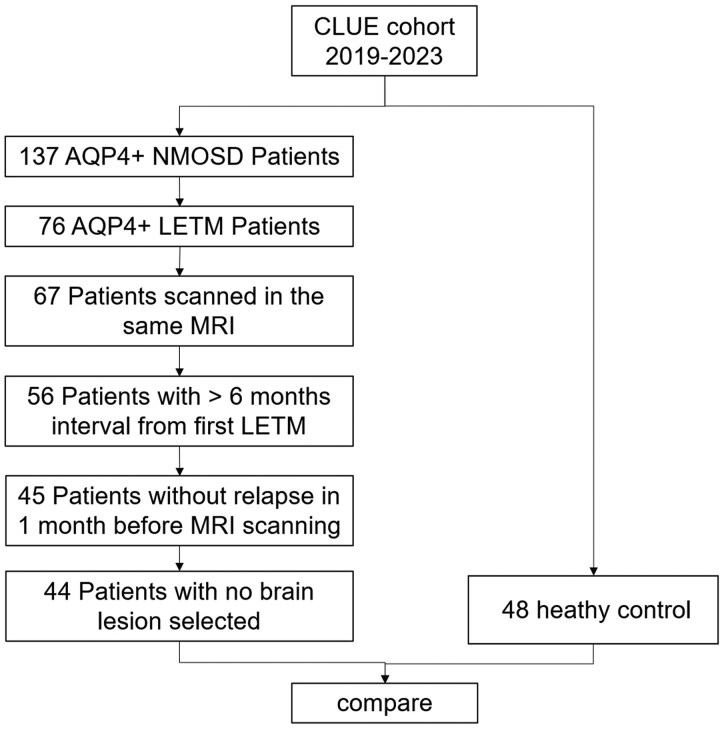
Flow diagram of the inclusion process.

173 patients diagnosed with AQP4-IgG + NMOSD and 48 healthy controls (HC) were screened from December 2018 to September 2023 in CLUE, an ongoing observational prospective cohort (NCT04106830). Inclusion criteria were (i) a definite diagnosis based on the 2015 international diagnostic criteria for NMOSD; (ii) seropositive AQP4-IgG determined by a cell-based assay (Kingmed, China); (iii) spinal cord lesions longer than three vertebral segments; (iv) receiving an MRI scan on the same scanner with HCs and (v) the scan interval longer than 6 months since the first LETM. Exclusion criteria included AQP4-IgG seronegativity, a relapse within one month prior to MRI, or presenting with short myelitis or brain lesions. The diagnosis and assessment were conducted under the supervision of two senior neuroimmunology specialists. All images were analysed by two experienced neuroimaging physicians. None of the patients had pyramidal disability prior to the LETM onset. In total, 92 MRI datasets from 44 AQP4+ LETM and 48 HC were analysed (Fig. 1).

### Clinical metrics and evaluation

Demographic and clinical characteristics of the patients were collected, including age, sex, disease duration, details of relapses and treatments, serum AQP4-IgG status and MRI examination date. MRI scan to the first LETM interval and annualized relapse rate were investigated. Spinal cord lesions were measured based on the most extensive LETM attack. Duration of pyramidal tract impairment was defined as the time interval between initial myelitis with pyramidal damage and MRI scan. The location and maximum length of LETM segments were determined using the cervical and thoracic spinal cord image with the total spinal cord lesion length. Expanded Disability Status Scale (EDSS) including pyramidal function score was recorded at the maximum LETM length to assess the clinical function for the patients. A re-evaluation of the EDSS was conducted, and a 25-Foot timed walk and a 9-Hole peg test were performed at the same time as the MRI scan. The age and sex imbalance between AQP4+ LETM and HC was adjusted as potentially confounding covariates in all statistical tests.

### Sample collection and analysis

Blood samples were obtained using clot-activating serum separator tubes, followed by centrifugation at 1000 g for 10 min to isolate serum. Subsequently, the serum was aliquoted and stored at −80°C until further analysis. Samples of AQP4+ LETM and HC that underwent initial magnetic resonance imaging scans within 1 month were analysed. Serum neurofilament light chain (NfL) and glial fibrillary acidic protein (GFAP) concentration were determined using the single-molecule arrays (SIMOA) Neuro 2-Plex B assay by Quanterix (Lexington, MA, USA).

### MRI data acquisition

All scans were acquired on a 3.0T Philips Ingenia CX scanner (Best, Netherlands) using a 48-channel head coil. Sagittal 3D FLAIR images were obtained with inversion recovery fast spin echo (IR-FSE) (TR/TE = 4800/228 ms, TI = 1650 ms, FA = 90°, 1 mm³ isotropic resolution, 196 slices), while sagittal 3D T1-weighted images employed MPRAGE (TR/TE = 6.6/3 ms, TI = 880 ms, FA = 8°, identical resolution). Diffusion imaging used axial multi-shell SE-EPI (TR/TE = 4000/88 ms, FA = 90°, 2.5 × 2.5 × 2.75 mm resolution, 60 slices) with *b*-values = 0/1000/2000s/mm² and 48 diffusion directions.

### Image analysis

#### Lesion segmentation

All the MR images were reviewed by an experienced neuroradiologist (Y.D. with more than 14 years’ experience in neuroradiology), and WMH were segmented using Lesion Segmentation Tool (version 3.0.0, https://www.applied-statistics.de/lst.html) using T1WI and FLAIR images. The segmented WMH was further checked and modified (if necessary) by a senior neuroradiologist (Y.D.). FLAIR and segmented WMH mask were normalized into the Montreal Neurological Institute space.

#### Diffusion image processing and metric calculation

The diffusion data were processed using MRtrix3.^18^ The dMRI images underwent denoising, eddy-current and motion correction, bias field correction and spatial resolution up-sampling to a voxel size of 1.25 mm³. After intensity normalization, fibre orientation distributions (FODs) were computed using multi-shell, three-tissue constrained spherical deconvolution (MS3T-CSD). Group specific template was generated from 40 subjects (20 NMOSD and 20 HC). Then, FOD images from each subject were subsequently registered to this template using FOD-guided non-linear registration.

To investigate the change in CST, we performed tract-of-interest analyses. We registered the seed ROIs for CST from the Johns Hopkins University atlas (provided in Supplementary material) to the population template using affine registration with antsRegistrationSyNQuick. Subsequently, a tractogram for CST was generated using probabilistic tractography on the population template. The 2000 streamlines with a 250 mm max length for CST tractogram generation were determined using the following parameters: ‘-maxlength 250-select 2000’ with tckgen in MRtrix3. A binary mask from the CST tractogram was used to extract relevant fixels. FBA was further analysed to provides quantitative measure, including fibre density (FD), fibre cross-section (FC) and fibre density and cross-section (FDC), of CST to assess both microstructural and macrostructural integrity.

Given that there were 96 slices of data available, further spatial analysis for CST was conducted by dividing these slices into 48 segments. Fixel-based metrics (FD, FC and FDC) were averaged across all fixels in each of the 48 CST segments. Fixels were selected based on the FOD peaks calculated using constrained spherical deconvolution. Details can be found in the Supplementary material code.

#### Statistical analysis

Statistical analyses were conducted using R version 4.3.1 (R Foundation for Statistical Computing, Vienna, Austria). Demographic characteristics were assessed using Wilcoxon rank-sum tests for continuous variables and χ^2^ tests for categorical variables between AQP4+ LETM and HC. MRI parameters including FD, FC and FDC of CST were assessed with linear regression in the two groups, correcting for age and sex. For the per-segment FBA analyses, we controlled for multiple comparisons using the Benjamini–Hochberg false discovery rate (FDR) correction method, with a significance threshold set at *P* < 0.05. In AQP4+ NMOSD patients with corticospinal cord involvement, FBA parameters of the CST were evaluated for their correlation with a range of clinical variables to identify the most clinically relevant predictors. Multivariate linear regression was performed to assess the relationships between FD, FDC and log_FC in the brain CST and three clinical measures: most severe pyramidal function score (CST score), time from pyramidal damage to MRI (CST time), and vertebral segments of spinal cord lesions at maximum LETM (CST segment). Age and sex were included as covariates, with multiple comparisons corrected. Partial correlation analysis was performed in FBA metrics of CST with biomarkers including NfL and GFAP, regressing out the effects of age and gender. A statistical significance threshold of two-sided *P* < 0.05 was adopted.

## Results

### Demographic and clinical characteristics

A total of 44 AQP4+ LETM (mean age 45.6 ± 14.2; 40 females [90.9%]) and 48 HC (mean age 41.8 ± 17.2; 34 females [70.8%]) were included in this study (Table 1). The AQP4+ LETM group exhibited a higher proportion of females compared to controls (*P* = 0.02). The median time interval between the initial NMOSD attack and MRI scanning was 3.8 (range 0.7–17.0) years. The median duration of pyramidal tract impairment was 3.1 (range 0.4–8.6) years. The median number of LETM attacks were 2.0 (1.0–5.0). Among the AQP4+ LETM patients, 52.3% had both cervical and thoracic spinal cord lesions, 29.5% had cervical lesions only, and 18.2% had isolated thoracic lesions. The median spinal cord lesion length on T2 were 7.0 (range 2.0–19.0) vertebral segments. The peak EDSS prior to MRI scanning was 5 (range 2.0–8.5), accompanied with a pyramidal function score of 3.0 (range 1.0–5.0). Median functional scores recorded at the time of the MRI scan included EDSS score 2.5 (range 0–8.0); 25-Foot timed walk second 5.3 (range 3.7–45.5); and 9-Hole peg test 19.9 (range 16.6–36.1) seconds. Rituximab was administered to 34 (77.3%) patients, while others received treatments include mycophenolate mofetil (6.8%), azathioprine (6.8%), cyclosporin (4.6%) and oral prednisone (2.3%).

**Table 1:** Demographic and clinical features of AQP4+ LETM patients and healthy controls

Characteristic	AQP4+ LETM (*n* = 44)	HC (*n* = 48)	*P*-value
Age, year^a^	45.6 ± 14.2	41.8 ± 17.2	0.19
Female^b^	40 (90.9)	34 (70.8)	0.02
Disease duration, year	3.8 (0.67–17.04)		
Pyramidal damage	39 (88.6)		
Pyramidal damage duration, year^c^	3.1 (0.4–8.6)		
LETM attacks	2.0 (1.0–5.0)		
CST attacks^c^	2.0(1.0–6.0)		
Immunosuppressive therapy^b^			
Rituximab	34 (77.3)		
Tocilizumab	3 (6.8)		
Mycophenolate mofetil	3 (6.8)		
Azathioprine	2 (4.6)		
Cyclosporin	1 (2.3)		
Prednisone	1 (2.3)		
Max-LETM measure			
Cervical^b^	13 (29.5)		
Thoracic^b^	8 (18.2)		
Cervical and thoracic^b^	24 (52.3)		
Total T2 length, vertebral segments	7.0 (2.0–19.0)		
Time interval, m			
Last LETM to MRI	11.5 (1.5–52.7)		
Maximum LETM to MRI	31.9 (4.5–111.1)		
Functional score			
Maximum LETM	4.5 (2–8.5)		
Worst EDSS	5 (2–8.5)		
EDSS at MRI scan	2.5 (0–8.0)		
Pyramidal function score^c^	3.0 (1.0–5.0)		
WHM volume, ml	0.19(0–7.02）		
25-Foot timed walk, s	5.3 (3.7–45.5)		
9-Hole peg test, s	19.9 (16.6–36.1)		
GFAP, pg/ml	154.0 (44.08–403.9)	72.2 (23.6–239.7)	0.00
NfL, pg/ml	16.5 (2.2–74.4)	7.1 (2.7–62.0)	0.00

Unless otherwise specified, data are medians, with ranges in parentheses.

LETM, longitudinally extensive transverse myelitis; AQP4+ LETM, AQP4-IgG + NMOSD a history of LETM before MRI scanning; EDSS, expanded disability status scale score; WHM, white matter hyperintensities; GFAP, glial fibrillary acidic protein; NfL, serum neurofilament light chain.

^a^Data are means ± SDs.

^b^Data are numbers of patients, with percentages in brackets.

^c^Only patients with pyramidal function damage were included.

### Fixel- and slice-wise analysis of CST

**Figure 2:**
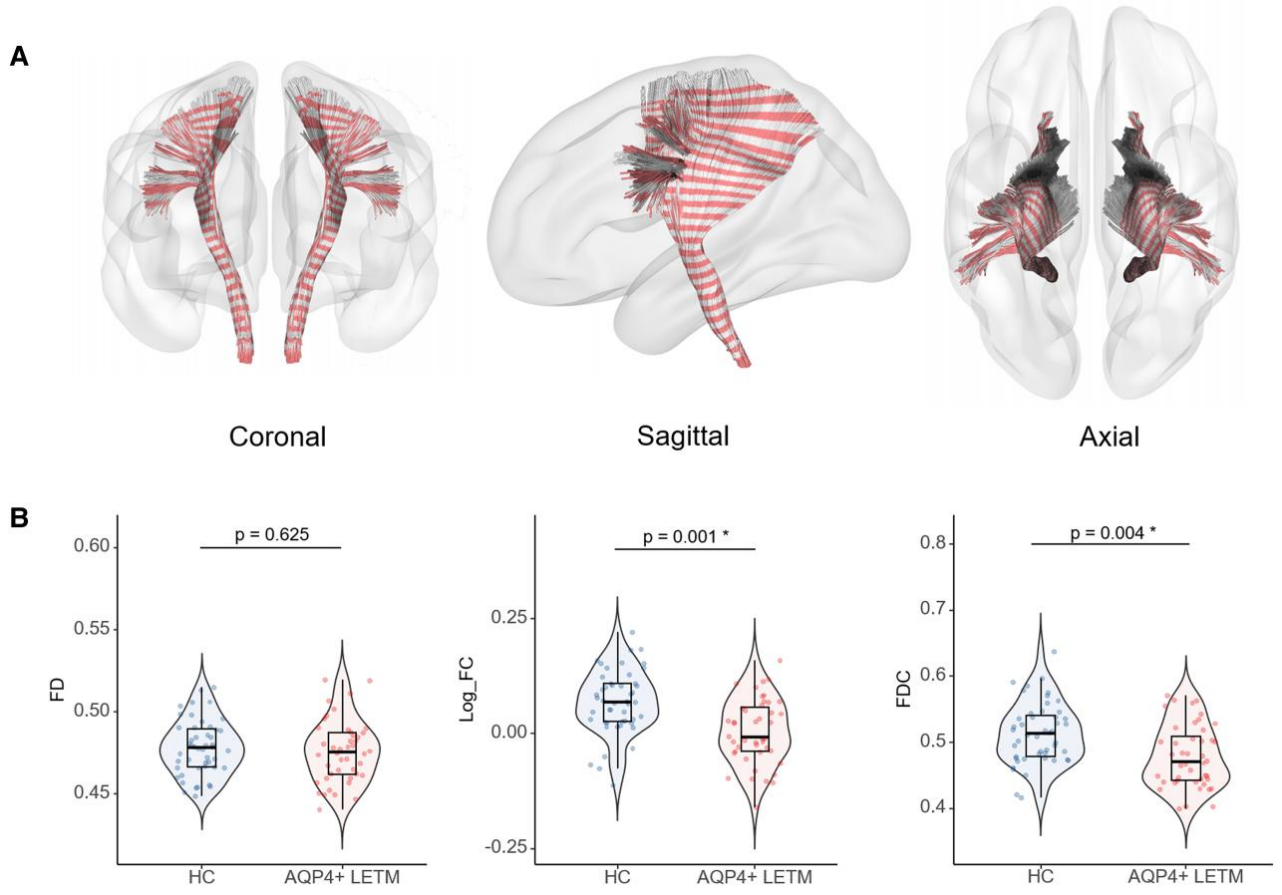
**Comparisons of mean CST values between AQP4+ LETM and HC.** (**A**) Coronal, Sagittal, and Axial views of the Pyramidal Tract with 48 Segments. (**B**) Comparison of mean values for FD, Log_FC and FDC in CST between AQP4+ LETM (right) and HC (left), FD, FC and FDC of the CST were assessed with linear regression, correcting for age and sex. Blue points represent HCs (*n* = 48), and red points represent AQP4+ LETM patients (AQP4 + LETM: *n* = 44). CST, corticospinal tract; AQP4, aquaporin-4; NMOSD, neuromyelitis optica spectrum disorder; HC, healthy control; FD, fibre density; FC, fibre-bundle cross-section; FDC, Log_FC * FD.

The CST in AQP4+ LETM patients exhibited significant macrostructural atrophy, with a reduction in log_FC (*P* = 0.001) compared to HCs. Reductions in the combined FDC metric (6.9% reduction, *P* = 0.004) were also observed in AQP4 + LETM. Microstructural differences (FD) were not significant between the two groups (Fig. 2).

**Figure 3:**
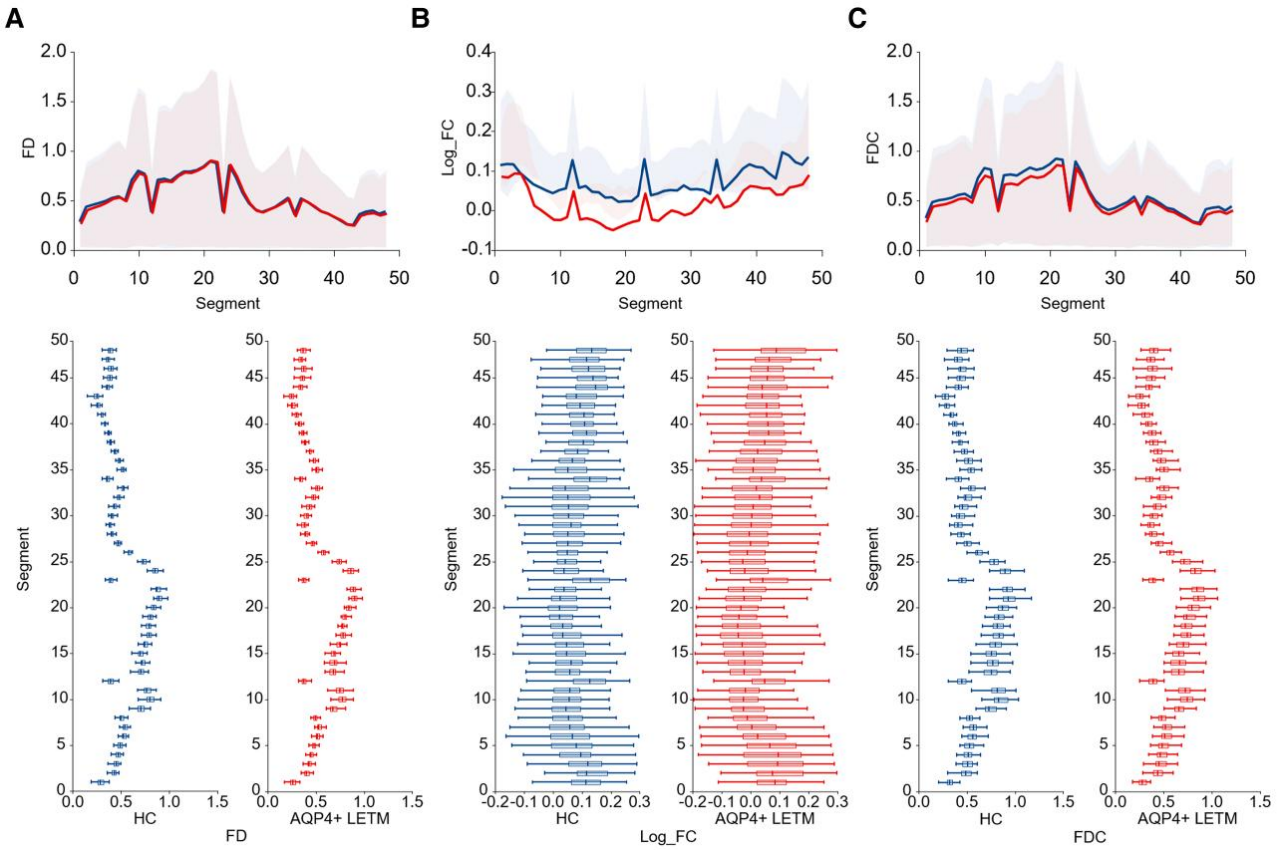
**Damage location of CST in AQP4+ LETM compared with HC.** Mean values for each of the 48 segments in CST of AQP4+ LETM (red) and HC (blue) for (**A**) FD, (**B**) Log_FC and (**C**) FDC. Segment 0 corresponds to the CST location closest to the spinal cord. Shading represents interquartile range and error bars are standard error. Per-segment FBA analyses were corrected for multiple comparisons using the Benjamini–Hochberg FDR method (*P* < 0.05). HC: *n* = 48; AQP4 + LETM: *n* = 44. CST, corticospinal tract; AQP4, aquaporin-4; NMOSD, neuromyelitis optica spectrum disorder; HC, healthy control; FD, fibre density; FC, fibre-bundle cross-section; FDC, Log_FC * FD.

In the 48 segments, combined by a total of 96 slices, 39 segments for log_FC exhibited statistically significant differences (*P* < 0.05; Supplementary Table 3; Fig. 3). The fixels demonstrating the greatest reduction in FDC were primarily located in the inferior part of CST. No significant differences were noted in FD and FDC across slices (Supplementary Tables 1 and 2; Fig. 3).

### Association of FBA metrics with clinical characteristics

**Figure 4:**
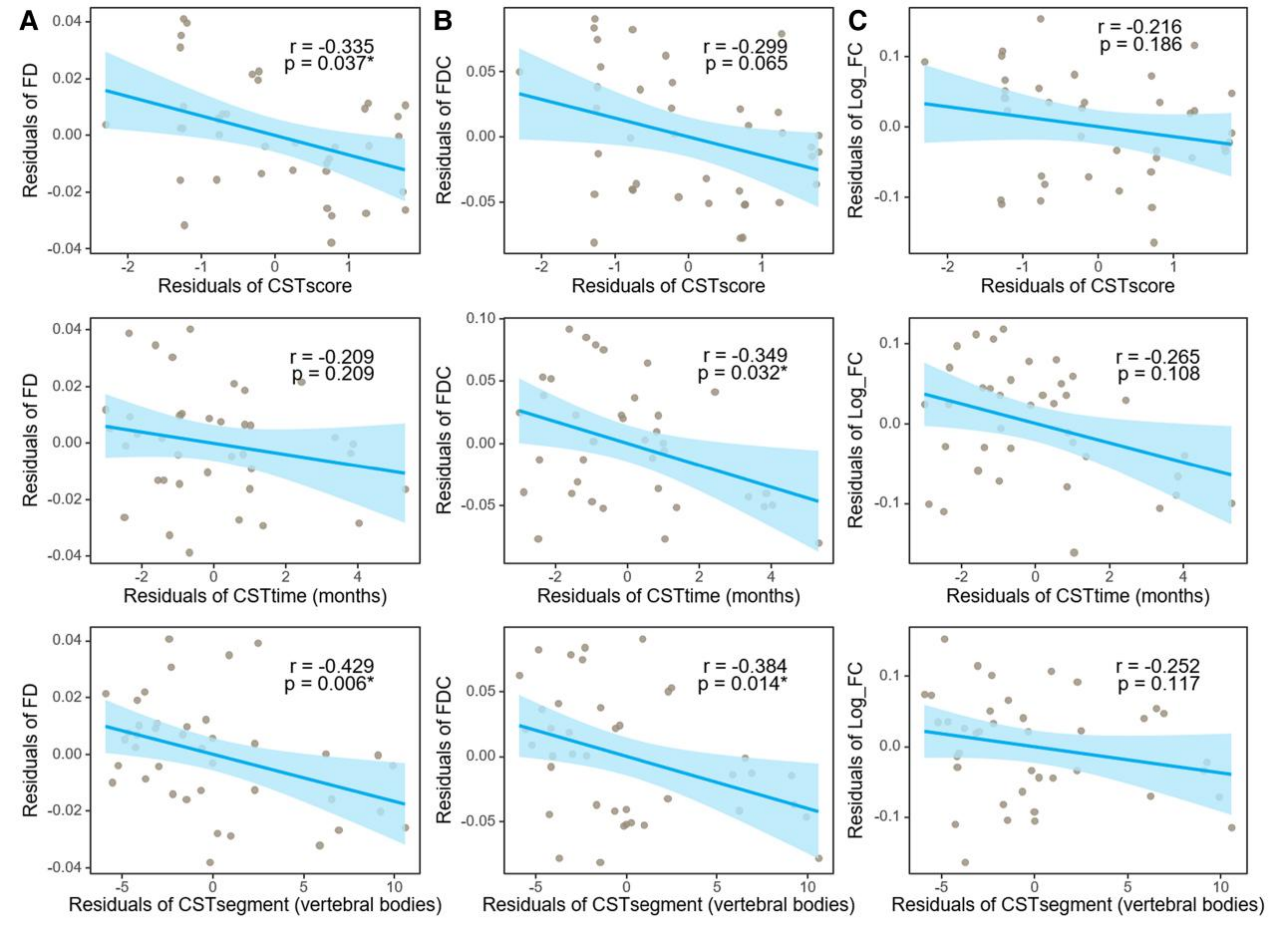
**Correlation between damage of brain CST and clinical features.** Scatterplots display the relationships between FD (**A**), FDC (**B**), and Log_FC (**C**) versus clinical measures (CST score/time/segment). Multivariate linear regression analysis was performed, including age and gender as covariates. Each point represents one AQP4+ LETM patient. The solid blue line represents the least-squares fit; the shaded area indicates the 95% confidence interval bounds of the slope; and the dashed blue lines denote the 95% prediction interval for new observations. Spearman’s correlation coefficients (**R**) And corresponding *P*-values are displayed in the upper right corner of each plot. Significance is indicated by an asterisk (*) for *P* < 0.05. *N* = 39. CST, corticospinal tract; FD, fibre density; FC, fibre-bundle cross-section; FDC, Log_FC * FD; CST score, pyramidal function score; CST time, months of pyramidal damage prior to MRI scan; CST segment, vertebral segments of spinal cord lesions at maximum LETM in patients with pyramidal function damage.

Correlation analysis showed that EDSS, pyramidal function score, and LETM segment correlated with FD; EDSS, pyramidal function score, LETM segment and spinal cord lesion-to-brain distance with FDC; and EDSS and spinal cord lesion-to-brain distance with log_FC (Supplementary Figs 1–3). Duration of pyramidal tract impairment, pyramidal function score and LETM segment emerged as significant predictors and were subsequently included in a multivariate linear regression model. After correction for age and sex, a significant negative association between FD and pyramidal function score (*r* = −0.335, *P* = 0.037) and LETM segment (*r* = −0.429, *P* = 0.006). Similarly, combined FDC metric demonstrated negative correlation with duration of pyramidal tract impairment (*r* = −0.349, *P* = 0.032) and LETM segment (*r* = −0.384, *P* = 0.014). There was no observed correlation between FC and any of these characteristics (Fig. 4). Whole model metrics for regression analysis were provided in Table 2.

**Table 2:** Model metrics for regression analysis

Target variable	Clinical variable	Adjusted *R*^2^	*F* statistic	*P*-value	Residual standard error
FD	CST score	0.138	7.062	0.012	0.018
FD	CST time	0.019	1.735	0.196	0.019
FD	CST segment	0.138	7.26	0.01	0.018
FDC	CST score	0.085	4.528	0.04	0.047
FDC	CST time	0.123	6.17	0.018	0.045
FDC	CST segment	0.123	6.45	0.015	0.046
Log_FC	CST score	0.02	1.788	0.189	0.074
Log_FC	CST time	0.103	5.228	0.028	0.067
Log_FC	CST segment	0.031	2.227	0.144	0.073

CST, corticospinal tract; FD, fibre density; FC, fibre-bundle cross-section; FDC, Log_FC * FD; CST score, pyramidal function score; CST time, months of pyramidal damage prior to MRI scan; CST segment, vertebral segments of spinal cord lesions at maximum LETM in patients with pyramidal function damage.

### Analysis of NfL and GFAP with FBA metrics

**Figure 5:**
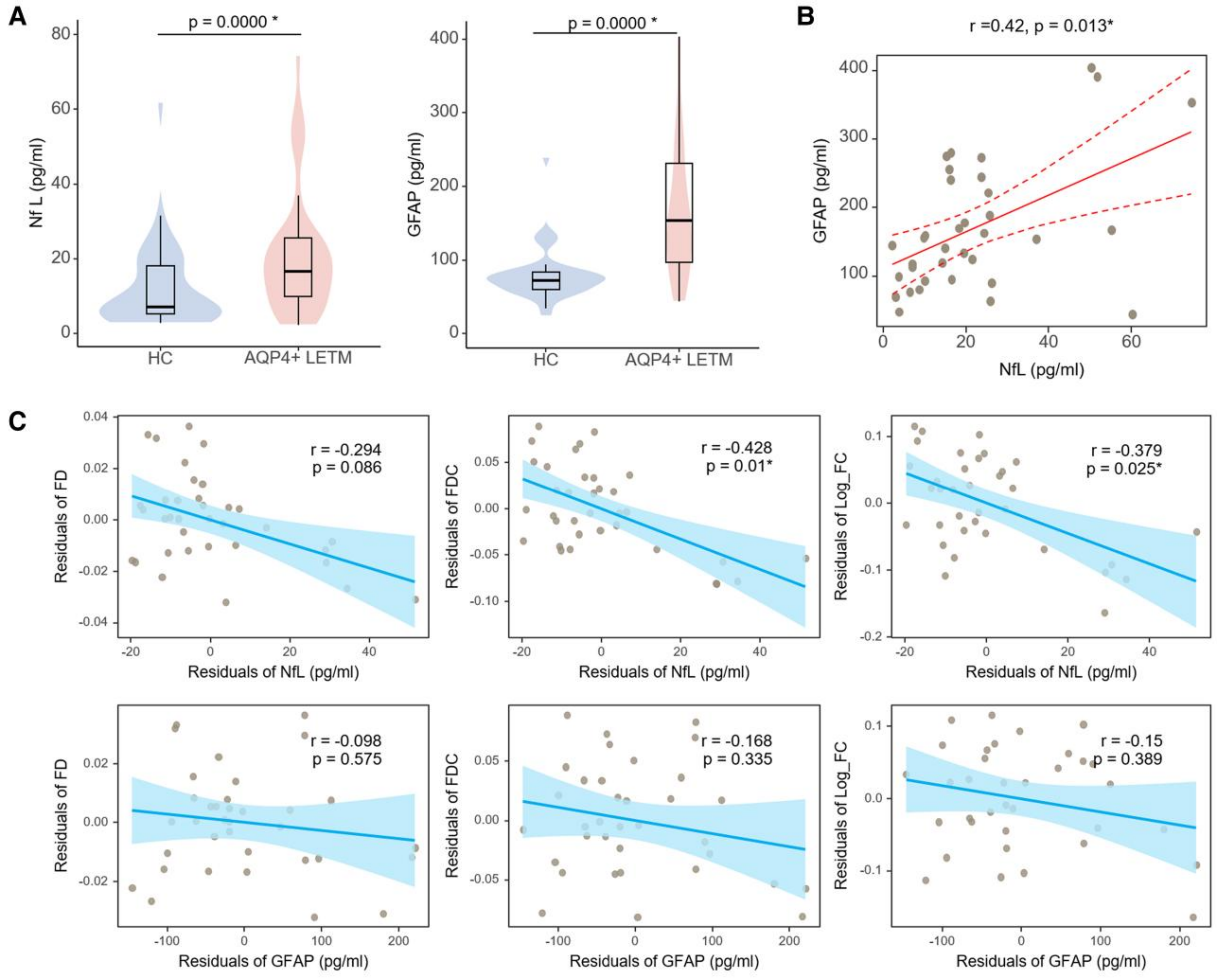
**Level of NfL and GFAP and association with FBA metrics.** (**A**) NfL and GFAP levels in AQP4+ LETM patients versus HC, adjusted for age and sex using linear regression. (**B**) Association between NfL and GFAP in AQP4+ LETM, assessed with Spearman’s correlation. (**C**) Partial correlations of NfL and GFAP with clinical measures (FD, Log_FC and FDC), regressing out age and sex effects. Residuals are plotted for visualization. In (**B**) and (**C**), each point represents an individual AQP4+ LETM patient. The solid blue line indicates the least-squares fit, with the shaded area showing the 95% confidence interval of the slope; dashed blue lines mark the 95% prediction interval for new observations. Spearman’s correlation coefficients (**R**) And *P*-values are annotated (upper right). Significance: *P* < 0.05 (*).HC: *n* = 37; AQP4+ LETM: *n* = 35. AQP4, aquaporin-4; NMOSD, neuromyelitis optica spectrum disorder; HC, healthy control; FD, fibre density; FC, fibre-bundle cross-section; FDC, Log_FC * FD; NfL, neurofilament light chain; GFAP, glial fibrillary acidic protein.

Serum NfL and GFAP levels were significantly increased in the AQP4 + LETM group than HC (NfL: 16.5 versus 7.1, *P* < 0.01; GFAP: 154.0 versus 72.2, *P* < 0.01) (Table 1 and Fig. 5). Additionally, a positive correlation was observed between GFAP and NfL levels in chronic phase of AQP4+ LETM (*r* = 0.42, *P* = 0.013).

Notably, during the chronic stage, NfL levels were found to be negatively correlated with FDC (*r* = −0. 428, *P* = 0.01) and log_FC (*r* = −0.379, *P* = 0.025), while NfL levels were not associated with FD. No significant associations were identified between GFAP levels and FD, FDC or log_FC during the chronic stage.

## Discussion

In the CLUE cohort, we explored alterations in the brain’s pyramidal tracts subsequent to long-segmental transverse myelitis in AQP-4-positive NMOSD patients. We identified axonal loss in the CST, particularly in regions closer to the spinal cord, as indicated by reductions in FC and FDC. Additionally, our results also revealed the clinical relevance of the structural changes, as associations were identified in CST metrics with the severity and duration of pyramidal impairment. We also noted negative association between FDC and NfL.

This is the first study to investigate the brain CST changes post AQP4+ LETM. The presence of damage to cerebral white matter in patients with NMOSD has been found by several studies.^11-14,19-21^ The application of FBA in this study represents a novel approach for quantifying white matter degeneration in NMOSD. Unlike conventional diffusion-weighted imaging techniques, FBA offers distinct advantages by mitigating crossing fibre effects and allowing the simultaneous analysis of FD and FC changes. Of note, our study focused on patients with AQP4+ LETM over six months and used FBA method to deal with the crossing fibre issues. The FC, measuring the cross-sectional area, captures macroscopic distinctions; meanwhile, the FD, reflecting fibre density, serves as an indicator of white matter microstructure, roughly correlating with total intra-axonal volume; the FDC, a composite of FC and FD, exhibits heightened sensitivity compared to either FD or FC in isolation.^18^ The observed reduction in FC and FDC, accompanied by the relative preservation of FD, indicates that the brain CST has undergone microscopic axonal loss, followed by a macroscopic collapse after six months of LETM.

Our slice-wise FBA revealed a significant spatial gradient, with the most pronounced changes in the inferior segments of the CST in AQP4+ LETM patients. Notably, patients in this study were selected without any brain lesions, thus our results emphasize the impact of LETM on the brain. Spatial gradient changes in CST, which indicates more severe damage in the section closer to the spinal cord lesion, have been identified in both spinal cord injury and a small sample study of NMOSD.^7,9,14^ Fractional anisotropy (FA) of CST were significantly reduced in the lower part of CST in 16 NMOSD.^12^ Our study enrolled more patients and overcame crossing fibre issues by using fixel-wise parameters with enhanced image resolution, offering a more integrated understanding of CST changes in AQP4+ LETM. These findings support the hypothesis that CST degeneration in NMOSD originates primarily from spinal cord damage through a retrodegenerative process. This process involves both axonal disconnection at the site of the spinal lesion and secondary degeneration of upstream structures. Unlike in MS, where demyelination predominates, CST degeneration in NMOSD appears to be more driven by axonal loss and subsequent retrograde degeneration, leading to progressive brain structural changes. Similarly, patients with LETM have a faster brain atrophy rate in NMOSD.^16^ These findings and our study both emphasize the impact of spinal cord damage on the upper brain.

The microstructural changes in CST may potentially be influenced by severity of pyramidal tract damage in spinal cord. In our study, a reduction in fibre density was associated both with the LETM segment length and pyramidal function score. The combined metric FDC, which integrates fibre cross-section and density, demonstrates a negative correlation with the LETM segment. The duration of pyramidal tract impairment may also affect the atrophy of CST. This is supported by a decrease in fibre cross-section and FDC with the duration of pyramidal damage increases, suggesting persistent damage in CST. In spinal cord injury, the tract derived from the motor cortex reorganizes variably due to different degrees of disruption of CST projections.^22^ This may explain the relation of CST changes with pyramidal tract damage. Besides, spinal cord injury could also lead to chronic brain neurodegeneration in animal models, this may explain the relationship of duration of pyramidal tract impairment and CST atrophy.^23^ Our results are also consistent with previous studies that have shown a correlation between disability in NMOSD and alterations in the upper motor pathway, including the CST or upper cervical area.^21,24-27^

In the chronic stage, elevated serum levels of NfL and GFAP in AQP4+ LETM patients, compared to HCs, suggest ongoing astrocytic damage, potentially contributing to CST atrophy. These biomarkers may serve as indicators of persistent neurodegeneration, even in the absence of clinical relapse. As NfL and GFAP are neuronal and astrocytic cytoskeletal proteins, our results indicated possible progressive axonal injury and damage of astrocyte in chronic phase of NMOSD. Ongoing subclinical increases in serum NfL and GFAP levels have also been reported in NMOSD previously.^28,29^ Meanwhile, NfL was negatively related to FDC and FC, higher NfL level accompanied with lower FDC and FC value in CST. The continuous damage in CST was supported by the decline in FDC and FC with the prolongation of the disease as well as the NfL elevation in chronic stage. A transcranial magnetic stimulation study on 10 stable NMOSD patients also revealed brain CST damage.^13^ It remains unclear whether the persistent damage was due to retrodegeneration, inflammatory involvement or silent progression of the disease. In many views, neurodegeneration is not considered to be absent between relapses,^30,31^ while others indicate possible silent progression based on electrophysiological, pathological and brain MRI volume analysis.^16,32,33^ Our study observed the damage in CST persisted into the chronic stage at least, which is suggestive of injury-related degeneration.

### Limitations

There are several limitations to this study. First, although we adjusted for age and sex in our analyses, potential confounding factors such as variability in treatment regimens and differences in disease duration prior to enrolment could still influence the observed results. Future longitudinal studies with larger cohorts are needed to fully disentangle these effects. Additionally, the study’s small sample size may have reduced statistical power in whole-brain fixel-based analyses, despite recruiting AQP-4 positive and LETM patients to minimize heterogeneity. Furthermore, our analysis focused exclusively on CST, and future studies should include analyses of additional white matter tracts (e.g. the corpus callosum, superior longitudinal fasciculus, and arcuate fasciculus) to confirm the specificity of these findings and to explore potential broader implications. Finally, our study’s findings on potential injury-related retrograde neurodegeneration in NMOSD remain limited, as the clinical relevance and disease progression implications are still unclear and require further validation.

## Conclusions

In summary, we demonstrate distinct brain CST morphological alterations in patients with AQP4+ LETM. Further, the brain pyramidal tracts alternation is related not only to the severity of myelitis, but also to the duration of the damage. The persistent elevation of NfL levels in the chronic phase of NMOSD, alongside CST atrophy, underscores the potential of NfL as a biomarker for tracking disease progression and guiding therapeutic interventions aimed at preventing ongoing neurodegeneration.

## Supplementary Material

fcaf257_Supplementary_Data

## Data Availability

The data generated and analysed during this study are available from the corresponding author upon reasonable request. The source code for specialized in-house scripts necessary for the reproduction of results has been uploaded as Supplementary material.
