# Supplementary material for: Brain corticospinal tract abnormalities in aquaporin-4 seropositive neuromyelitis optica spectrum disorder with longitudinally extensive transverse myelitis
Source: Brain Commun. 2025 Jun 27;7(4):fcaf257. doi: 10.1093/braincomms/fcaf257 (PMC12342178; doi:10.1093/braincomms/fcaf257)
Supplement: fcaf257_Supplementary_Data [file fcaf257_supplementary_data.docx]

**Supplementary Material**

Title: *Brain Corticospinal Tract Abnormalities in Aquaporin-4 Seropositive NMOSD with Longitudinally Extensive Transverse Myelitis*

**Content**

**Supplement Table 1 Segment Differences in FD**

**Supplement Table 2 Segment Differences in FDC**

**Supplement Table 3 Segment Differences in Log_FC**

**Supplement Figure 1: Associations between FD and Clinical Parameters (Age- and Gender-Adjusted)**

**Supplement Figure 2: Associations between FDC and Clinical Parameters (Age- and Gender-Adjusted)**

**Supplement Figure 3: Associations between FDC and Clinical Parameters (Age- and Gender-Adjusted)**

**Code used in the manuscript**

**Supplement Table 1 Segment Differences in FD**

| **Segment** | **Estimated Difference in FD** | **Standard Error** | **Statistic** | **P value** | **Adjusted P** |
| --- | --- | --- | --- | --- | --- |
| 1 | -0.01322 | 0.010018 | -1.31965 | 0.190376 | 0.397307 |
| 2 | -0.01296 | 0.007694 | -1.68424 | 0.095679 | 0.353276 |
| 3 | -0.01329 | 0.006941 | -1.91474 | 0.058774 | 0.235097 |
| 4 | -0.01237 | 0.006226 | -1.98611 | 0.050134 | 0.235097 |
| 5 | -0.01551 | 0.006325 | -2.45267 | 0.016153 | 0.235097 |
| 6 | -0.01617 | 0.007734 | -2.09055 | 0.039453 | 0.235097 |
| 7 | -0.01597 | 0.00828 | -1.92902 | 0.056951 | 0.235097 |
| 8 | -0.01082 | 0.007741 | -1.39815 | 0.165583 | 0.378476 |
| 9 | -0.01536 | 0.007743 | -1.98363 | 0.050415 | 0.235097 |
| 10 | -0.01425 | 0.00729 | -1.9547 | 0.053793 | 0.235097 |
| 11 | -0.01569 | 0.007016 | -2.23682 | 0.027826 | 0.235097 |
| 12 | -0.01415 | 0.006443 | -2.1955 | 0.030759 | 0.235097 |
| 13 | -0.00937 | 0.006439 | -1.45451 | 0.149363 | 0.364455 |
| 14 | -0.00453 | 0.006488 | -0.6984 | 0.486766 | 0.753702 |
| 15 | -0.00305 | 0.006337 | -0.48205 | 0.630969 | 0.839744 |
| 16 | -0.00528 | 0.006345 | -0.832 | 0.407661 | 0.673942 |
| 17 | -0.01135 | 0.010437 | -1.08792 | 0.2796 | 0.536831 |
| 18 | -0.01587 | 0.011844 | -1.33956 | 0.183837 | 0.397307 |
| 19 | -0.01697 | 0.010826 | -1.56739 | 0.120613 | 0.364455 |
| 20 | -0.02279 | 0.00943 | -2.41714 | 0.017707 | 0.235097 |
| 21 | -0.02727 | 0.009744 | -2.7984 | 0.006309 | 0.235097 |
| 22 | -0.01604 | 0.007871 | -2.03743 | 0.04461 | 0.235097 |
| 23 | -0.01196 | 0.007908 | -1.51305 | 0.133851 | 0.364455 |
| 24 | -0.0071 | 0.007535 | -0.94202 | 0.348761 | 0.643866 |
| 25 | -0.0054 | 0.006412 | -0.84211 | 0.40201 | 0.673942 |
| 26 | -0.00095 | 0.00619 | -0.15301 | 0.878739 | 0.930575 |
| 27 | 0.003282 | 0.006625 | 0.495363 | 0.621579 | 0.839744 |
| 28 | 0.006684 | 0.007366 | 0.907438 | 0.366654 | 0.651829 |
| 29 | 0.008469 | 0.007486 | 1.131324 | 0.260992 | 0.521985 |
| 30 | 0.012391 | 0.00778 | 1.592565 | 0.114844 | 0.364455 |
| 31 | 0.010332 | 0.006778 | 1.524368 | 0.131003 | 0.364455 |
| 32 | 0.003467 | 0.005372 | 0.645464 | 0.520306 | 0.768323 |
| 33 | -0.00231 | 0.005023 | -0.45909 | 0.647302 | 0.839744 |
| 34 | -0.00772 | 0.005339 | -1.44555 | 0.151856 | 0.364455 |
| 35 | -0.0084 | 0.005626 | -1.49237 | 0.139177 | 0.364455 |
| 36 | -0.00489 | 0.00605 | -0.8081 | 0.421214 | 0.673942 |
| 37 | -0.00086 | 0.006308 | -0.13642 | 0.891801 | 0.930575 |
| 38 | -0.000908 | 0.006104 | -0.14878 | 0.882067 | 0.930575 |
| 39 | -0.002 | 0.005826 | -0.34309 | 0.732349 | 0.901352 |
| 40 | -0.00271 | 0.005008 | -0.54118 | 0.589753 | 0.832592 |
| 41 | -0.00111 | 0.004564 | -0.24262 | 0.808864 | 0.930575 |
| 42 | 2.98E-06 | 0.004221 | 7.06E-04 | 0.999439 | 0.999439 |
| 43 | -0.00115 | 0.004079 | -0.28081 | 0.779512 | 0.930575 |
| 44 | -0.00098 | 0.004279 | -0.23009 | 0.818556 | 0.930575 |
| 45 | -0.00044 | 0.004364 | -0.10133 | 0.919517 | 0.939081 |
| 46 | 8.29E-04 | 0.00483 | 0.171638 | 0.864116 | 0.930575 |
| 47 | 0.002118 | 0.005647 | 0.375123 | 0.708472 | 0.894911 |
| 48 | 0.004834 | 0.007634 | 0.633235 | 0.528222 | 0.768323 |

**Supplement Table 2 Segment Differences in FDC**

| **Segment** | **Estimated Difference in FD** | **Standard Error** | **Statistic** | **P value** | **Adjusted P** |
| --- | --- | --- | --- | --- | --- |
| 1 | -0.01322 | 0.010018 | -1.31965 | 0.190376 | 0.397307 |
| 2 | -0.01296 | 0.007694 | -1.68424 | 0.095679 | 0.353276 |
| 3 | -0.01329 | 0.006941 | -1.91474 | 0.058774 | 0.235097 |
| 4 | -0.01237 | 0.006226 | -1.98611 | 0.050134 | 0.235097 |
| 5 | -0.01551 | 0.006325 | -2.45267 | 0.016153 | 0.235097 |
| 6 | -0.01617 | 0.007734 | -2.09055 | 0.039453 | 0.235097 |
| 7 | -0.01597 | 0.00828 | -1.92902 | 0.056951 | 0.235097 |
| 8 | -0.01082 | 0.007741 | -1.39815 | 0.165583 | 0.378476 |
| 9 | -0.01536 | 0.007743 | -1.98363 | 0.050415 | 0.235097 |
| 10 | -0.01425 | 0.00729 | -1.9547 | 0.053793 | 0.235097 |
| 11 | -0.01569 | 0.007016 | -2.23682 | 0.027826 | 0.235097 |
| 12 | -0.01415 | 0.006443 | -2.1955 | 0.030759 | 0.235097 |
| 13 | -0.00937 | 0.006439 | -1.45451 | 0.149363 | 0.364455 |
| 14 | -0.00453 | 0.006488 | -0.6984 | 0.486766 | 0.753702 |
| 15 | -0.00305 | 0.006337 | -0.48205 | 0.630969 | 0.839744 |
| 16 | -0.00528 | 0.006345 | -0.832 | 0.407661 | 0.673942 |
| 17 | -0.01135 | 0.010437 | -1.08792 | 0.2796 | 0.536831 |
| 18 | -0.01587 | 0.011844 | -1.33956 | 0.183837 | 0.397307 |
| 19 | -0.01697 | 0.010826 | -1.56739 | 0.120613 | 0.364455 |
| 20 | -0.02279 | 0.00943 | -2.41714 | 0.017707 | 0.235097 |
| 21 | -0.02727 | 0.009744 | -2.7984 | 0.006309 | 0.235097 |
| 22 | -0.01604 | 0.007871 | -2.03743 | 0.04461 | 0.235097 |
| 23 | -0.01196 | 0.007908 | -1.51305 | 0.133851 | 0.364455 |
| 24 | -0.0071 | 0.007535 | -0.94202 | 0.348761 | 0.643866 |
| 25 | -0.0054 | 0.006412 | -0.84211 | 0.40201 | 0.673942 |
| 26 | -0.000947 | 0.00619 | -0.15301 | 0.878739 | 0.930575 |
| 27 | 0.003282 | 0.006625 | 0.495363 | 0.621579 | 0.839744 |
| 28 | 0.006684 | 0.007366 | 0.907438 | 0.366654 | 0.651829 |
| 29 | 0.008469 | 0.007486 | 1.131324 | 0.260992 | 0.521985 |
| 30 | 0.012391 | 0.00778 | 1.592565 | 0.114844 | 0.364455 |
| 31 | 0.010332 | 0.006778 | 1.524368 | 0.131003 | 0.364455 |
| 32 | 0.003467 | 0.005372 | 0.645464 | 0.520306 | 0.768323 |
| 33 | -0.00231 | 0.005023 | -0.45909 | 0.647302 | 0.839744 |
| 34 | -0.00772 | 0.005339 | -1.44555 | 0.151856 | 0.364455 |
| 35 | -0.0084 | 0.005626 | -1.49237 | 0.139177 | 0.364455 |
| 36 | -0.00489 | 0.00605 | -0.8081 | 0.421214 | 0.673942 |
| 37 | -0.00086 | 0.006308 | -0.13642 | 0.891801 | 0.930575 |
| 38 | -0.00091 | 0.006104 | -0.14878 | 0.882067 | 0.930575 |
| 39 | -0.002 | 0.005826 | -0.34309 | 0.732349 | 0.901352 |
| 40 | -0.00271 | 0.005008 | -0.54118 | 0.589753 | 0.832592 |
| 41 | -0.00111 | 0.004564 | -0.24262 | 0.808864 | 0.930575 |
| 42 | 2.98E-06 | 0.004221 | 7.06E-04 | 0.999439 | 0.999439 |
| 43 | -0.00115 | 0.004079 | -0.28081 | 0.779512 | 0.930575 |
| 44 | -0.00098 | 0.004279 | -0.23009 | 0.818556 | 0.930575 |
| 45 | -0.00044 | 0.004364 | -0.10133 | 0.919517 | 0.939081 |
| 46 | 8.29E-04 | 0.00483 | 0.171638 | 0.864116 | 0.930575 |
| 47 | 0.002118 | 0.005647 | 0.375123 | 0.708472 | 0.894911 |
| 48 | 0.004834 | 0.007634 | 0.633235 | 0.528222 | 0.768323 |

**Supplement Table 3 Segment Differences in Log_FC**

| **Segment** | **Estimated Difference in FD** | **Standard Error** | **Statistic** | **P value** | **Adjusted P** |
| --- | --- | --- | --- | --- | --- |
| 1 | -0.03583 | 0.015564 | -2.3022 | 0.023684 | 0.031578 |
| 2 | -0.06157 | 0.017225 | -3.57443 | 5.73E-04 | 0.001963 |
| 3 | -0.06731 | 0.017306 | -3.88969 | 1.95E-04 | 0.001037 |
| 4 | -0.06975 | 0.017547 | -3.97498 | 1.44E-04 | 8.63E-04 |
| 5 | -0.07139 | 0.017816 | -4.0069 | 1.28E-04 | 8.63E-04 |
| 6 | -0.06125 | 0.018042 | -3.39489 | 0.001032 | 0.002476 |
| 7 | -0.04637 | 0.017736 | -2.6147 | 0.010506 | 0.014837 |
| 8 | -0.02846 | 0.016886 | -1.68536 | 0.095462 | 0.109099 |
| 9 | -0.02233 | 0.020574 | -1.08527 | 0.280766 | 0.30629 |
| 10 | -0.01601 | 0.022273 | -0.71874 | 0.474203 | 0.49482 |
| 11 | -0.01066 | 0.023537 | -0.45275 | 0.651846 | 0.651846 |
| 12 | -0.01415 | 0.025422 | -0.55656 | 0.579241 | 0.591565 |
| 13 | -0.02521 | 0.027105 | -0.9301 | 0.354863 | 0.378521 |
| 14 | -0.03727 | 0.027103 | -1.37504 | 0.17261 | 0.192681 |
| 15 | -0.04411 | 0.024398 | -1.80788 | 0.074042 | 0.086684 |
| 16 | -0.04548 | 0.019957 | -2.27875 | 0.025102 | 0.032565 |
| 17 | -0.05729 | 0.019656 | -2.91462 | 0.004515 | 0.006991 |
| 18 | -0.06455 | 0.018937 | -3.40841 | 9.88E-04 | 0.002476 |
| 19 | -0.06649 | 0.01737 | -3.82751 | 2.42E-04 | 0.001055 |
| 20 | -0.0685 | 0.015431 | -4.43903 | 2.61E-05 | 2.51E-04 |
| 21 | -0.07724 | 0.015734 | -4.90875 | 4.18E-06 | 1.05E-04 |
| 22 | -0.08058 | 0.016453 | -4.89755 | 4.37E-06 | 1.05E-04 |
| 23 | -0.07992 | 0.01719 | -4.64907 | 1.17E-05 | 1.86E-04 |
| 24 | -0.07691 | 0.016955 | -4.53605 | 1.80E-05 | 2.16E-04 |
| 25 | -0.07182 | 0.017032 | -4.2165 | 6.00E-05 | 4.80E-04 |
| 26 | -0.06564 | 0.017073 | -3.84469 | 2.28E-04 | 0.001055 |
| 27 | -0.06158 | 0.01716 | -3.58856 | 5.46E-04 | 0.001963 |
| 28 | -0.0602 | 0.017528 | -3.43476 | 9.07E-04 | 0.002418 |
| 29 | -0.05943 | 0.017598 | -3.37702 | 0.001093 | 0.002498 |
| 30 | -0.06084 | 0.017423 | -3.49177 | 7.53E-04 | 0.002245 |
| 31 | -0.05428 | 0.016502 | -3.28924 | 0.001446 | 0.003154 |
| 32 | -0.05252 | 0.016657 | -3.15306 | 0.00221 | 0.004243 |
| 33 | -0.05444 | 0.017435 | -3.12257 | 0.002427 | 0.00448 |
| 34 | -0.05761 | 0.018542 | -3.10705 | 0.002544 | 0.0045 |
| 35 | -0.05853 | 0.019409 | -3.0157 | 0.003351 | 0.005546 |
| 36 | -0.05909 | 0.019081 | -3.09672 | 0.002625 | 0.0045 |
| 37 | -0.05658 | 0.018895 | -2.99452 | 0.003569 | 0.00571 |
| 38 | -0.04999 | 0.019118 | -2.61457 | 0.01051 | 0.014837 |
| 39 | -0.043 | 0.019932 | -2.15761 | 0.033683 | 0.042547 |
| 40 | -0.03929 | 0.019806 | -1.98368 | 0.050408 | 0.06049 |
| 41 | -0.03895 | 0.018722 | -2.08059 | 0.040379 | 0.049697 |
| 42 | -0.04178 | 0.01737 | -2.40516 | 0.01826 | 0.025042 |
| 43 | -0.04611 | 0.016429 | -2.80679 | 0.006161 | 0.009241 |
| 44 | -0.05092 | 0.015712 | -3.24077 | 0.001684 | 0.003514 |
| 45 | -0.05343 | 0.015143 | -3.5281 | 6.68E-04 | 0.002137 |
| 46 | -0.05519 | 0.015117 | -3.65068 | 4.43E-04 | 0.001773 |
| 47 | -0.05279 | 0.01519 | -3.47496 | 7.95E-04 | 0.002245 |
| 48 | -0.05157 | 0.01616 | -3.19141 | 0.001963 | 0.003927 |

**Supplement Figure 1: Associations between FD and Clinical Parameters (Age- and Gender-Adjusted)
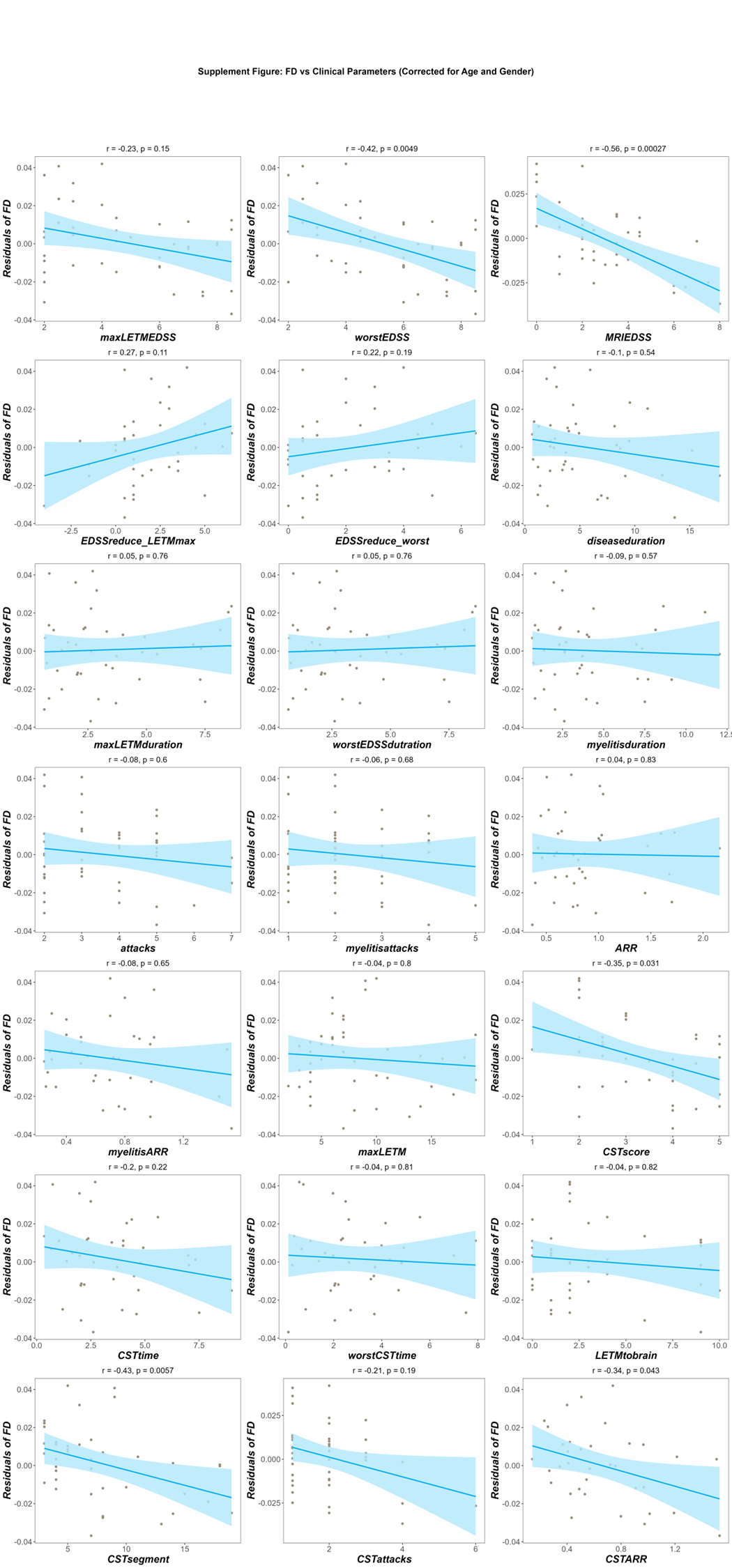
**

**Supplement Figure 2: Associations between FDC and Clinical Parameters (Age- and Gender-Adjusted)**
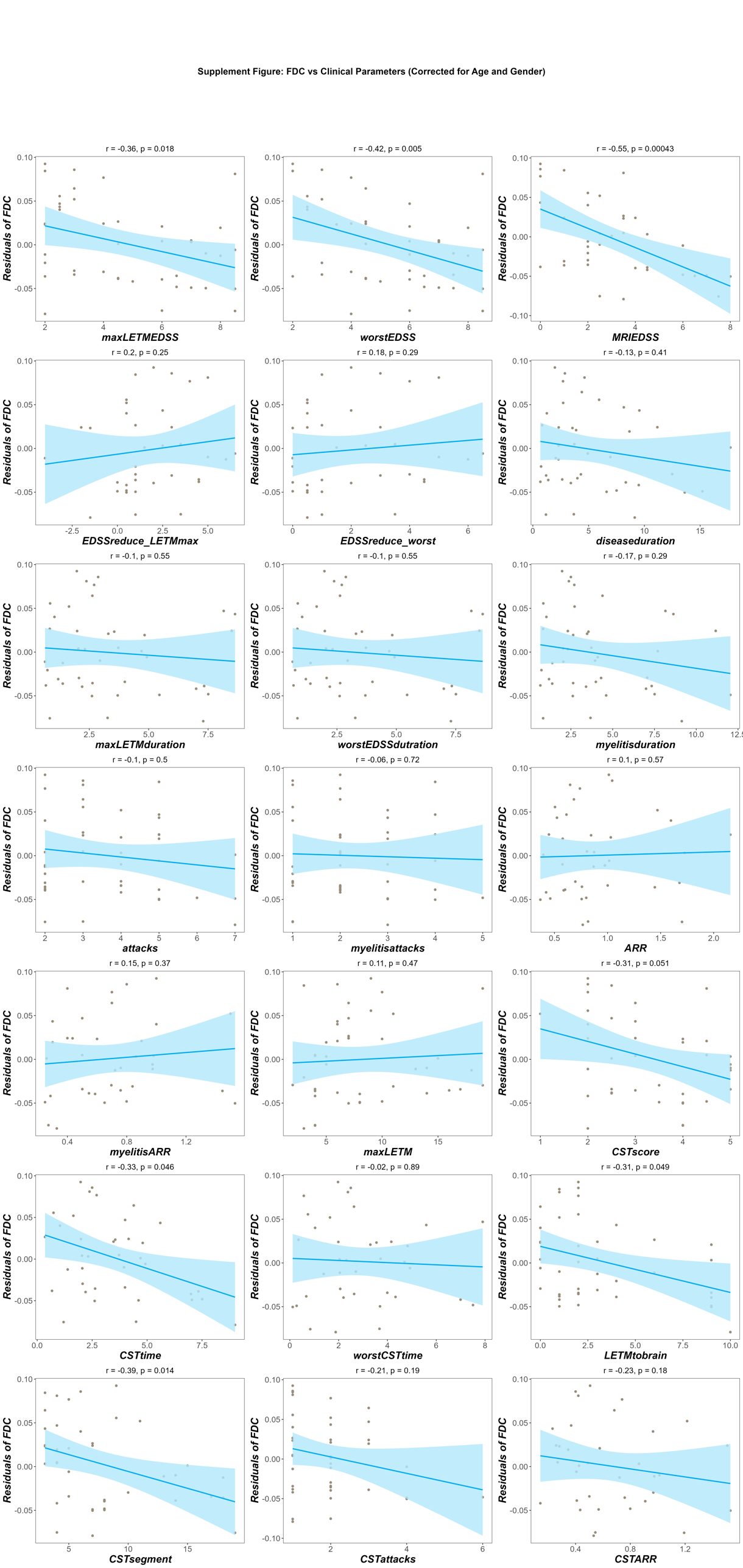


**Supplement Figure 3: Associations between Log_FC and Clinical Parameters (Age- and Gender-Adjusted)**
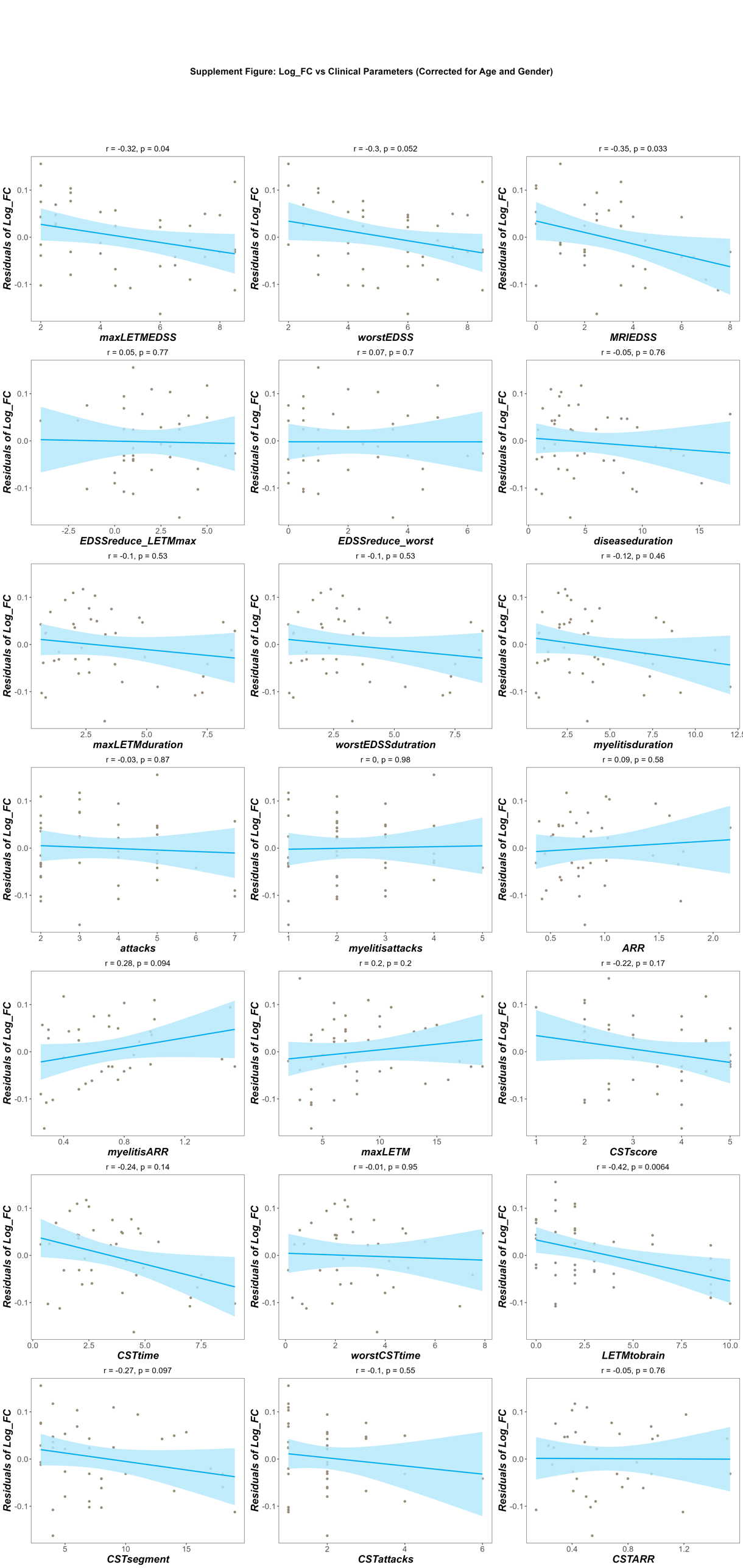


**Code used in the manuscript**

for_each * : mrconvert IN/DKI.nii.gz IN/DKI.mif -fslgrad IN/DKI.bvec IN/DKI.bval

***#Denoising and unringing***

for_each * : dwidenoise IN/DKI.mif IN/dwi_denoised.mif

for_each * : mrdegibbs IN/dwi_denoised.mif IN/dwi_denoised_unringed.mif -axes 0,1

***#Motion and distortion correction***

for_each * : dwifslpreproc IN/dwi_denoised_unringed.mif IN/dwi_denoised_unringed_preproc.mif -rpe_none -pe_dir AP

***#Bias field correction***

for_each * : dwibiascorrect ants IN/dwi_denoised_unringed_preproc.mif IN/dwi_denoised_unringed_preproc_unbiased.mif

***#Computing (average) tissue response functions***

for_each * : dwi2response dhollander IN/dwi_denoised_unringed_preproc_unbiased.mif IN/response_wm.txt IN/response_gm.txt IN/response_csf.txt

responsemean */response_wm.txt ../group_average_response_wm.txt -force

responsemean */response_gm.txt ../group_average_response_gm.txt -force

responsemean */response_csf.txt ../group_average_response_csf.txt -force

***#Upsampling DW images***

for_each * : mrgrid IN/dwi_denoised_unringed_preproc_unbiased.mif regrid -vox 1.25 IN/dwi_denoised_unringed_preproc_unbiased_upsampled.mif -force

***#Compute upsampled brain mask images***

for_each * : mrconvert IN/dwi_denoised_unringed_preproc_unbiased_upsampled.mif IN/dwi_denoised_unringed_preproc_unbiased_upsampled.nii.gz

for_each * : fslroi IN/dwi_denoised_unringed_preproc_unbiased_upsampled.nii.gz IN/dwi_denoised_unringed_preproc_unbiased_upsampled_extracted.nii.gz 0 1

for_each * : mri_synthstrip -i IN/dwi_denoised_unringed_preproc_unbiased_upsampled_extracted.nii.gz -o IN/dwi_mask_upsampled_synthstriped.nii.gz -m IN/mask.nii.gz -force

for_each * : mrconvert --datatype uint8 IN/mask.nii.gz IN/dwi_mask_upsampled_2.mif

***#Fibre Orientation Distribution estimation (multi-tissue spherical deconvolution)***

for_each * : dwi2fod msmt_csd IN/dwi_denoised_unringed_preproc_unbiased_upsampled.mif ../group_average_response_wm.txt IN/wmfod.mif ../group_average_response_gm.txt IN/gm.mif ../group_average_response_csf.txt IN/csf.mif -mask IN/dwi_mask_upsampled_2.mif

***#Joint bias field correction and intensity normalisation***

for_each * : mtnormalise IN/wmfod.mif IN/wmfod_norm.mif IN/gm.mif IN/gm_norm.mif IN/csf.mif IN/csf_norm.mif -mask IN/dwi_mask_upsampled_2.mif

***#Generate a study-specific unbiased FOD template***

mkdir -p ../template/fod_input

mkdir ../template/mask_input

for_each `ls -d P* | head -20` : ln -sr IN/wmfod_norm.mif ../template/fod_input/PRE.mif ";" ln -sr IN/dwi_mask_upsampled_2.mif ../template/mask_input/PRE.mif

for_each `ls -d T* | head -20` : ln -sr IN/wmfod_norm.mif ../template/fod_input/PRE.mif ";" ln -sr IN/dwi_mask_upsampled_2.mif ../template/mask_input/PRE.mif

population_template ../template/fod_input -mask_dir ../template/mask_input ../template/wmfod_template.mif -voxel_size 1.25

for_each * : mrregister IN/wmfod_norm.mif -mask1 IN/dwi_mask_upsampled_2.mif ../template/wmfod_template.mif -nl_warp IN/subject2template_warp.mif IN/template2subject_warp.mif

***#Compute template_mask***

for_each * : mrtransform IN/dwi_mask_upsampled_2.mif -warp IN/subject2template_warp.mif -interp nearest -datatype bit IN/dwi_mask_in_template_space.mif

mrmath */dwi_mask_in_template_space.mif min ../template/template_mask.mif -datatype bit

mkdir -p ../test/fod_input

mkdir ../test/mask_input

for_each `ls -d A* | head -22` : ln -sr IN/wmfod_norm_transformed.mif ../test/fod_input/PRE.mif ";" ln -sr IN/dwi_mask_in_template_space.mif ../test/mask_input/PRE.mif

for_each `ls -d B* | head -22` : ln -sr IN/wmfod_norm_transformed.mif ../test/fod_input/PRE.mif ";" ln -sr IN/dwi_mask_in_template_space.mif ../test/mask_input/PRE.mif

fod2fixel -mask ../template/template_mask.mif -fmls_peak_value 0.06 ../template/wmfod_template.mif ../template/fixel_mask

mrinfo -size ../template/fixel_mask/directions.mif

for_each * : mrtransform IN/wmfod_norm.mif -warp IN/subject2template_warp.mif -reorient_fod no IN/fod_in_template_space_NOT_REORIENTED.mif

for_each * : fod2fixel -mask ../template/template_mask.mif IN/fod_in_template_space_NOT_REORIENTED.mif IN/fixel_in_template_space_NOT_REORIENTED -afd fd.mif

for_each * : fixelreorient IN/fixel_in_template_space_NOT_REORIENTED IN/subject2template_warp.mif IN/fixel_in_template_space

for_each * : fixelcorrespondence IN/fixel_in_template_space/fd.mif ../template/fixel_mask ../template/fd PRE.mif

for_each * : warp2metric IN/subject2template_warp.mif -fc ../template/fixel_mask ../template/fc IN.mif

mkdir ../template/log_fc

cp ../template/fc/index.mif ../template/fc/directions.mif ../template/log_fc

for_each * : mrcalc ../template/fc/IN.mif -log ../template/log_fc/IN.mif

mkdir ../template/fdc

cp ../template/fc/index.mif ../template/fdc

cp ../template/fc/directions.mif ../template/fdc

for_each * : mrcalc ../template/fd/IN.mif ../template/fc/IN.mif -mult ../template/fdc/IN.mif

cd ../template

tckgen -angle 22.5 -maxlen 250 -minlen 10 -power 1.0 wmfod_template.mif -seed_image template_mask.mif -mask template_mask.mif -select 20000000 -cutoff 0.06 tracks_20_million.tck

tcksift tracks_20_million.tck wmfod_template.mif tracks_2_million_sift.tck -term_number 2000000

fixelconnectivity fixel_mask/ tracks_2_million_sift.tck matrix/

fixelfilter fd smooth fd_smooth -matrix matrix/

fixelfilter log_fc smooth log_fc_smooth -matrix matrix/

fixelfilter fdc smooth fdc_smooth -matrix matrix/

***##statistics for whole brain***

fixelcfestats fd_smooth/ files.txt design_matrix.txt contrast_matrix.txt matrix/ stats_fd/

fixelcfestats log_fc_smooth/ files.txt design_matrix.txt contrast_matrix.txt matrix/ stats_fc/

fixelcfestats fdc_smooth/ files.txt design_matrix.txt contrast_matrix.txt matrix/ stats_fdc/

***#register the FA template in standard space to wmfod***

mrconvert wmfod_template.mif wmfod_template.nii.gz

fslroi wmfod_template.nii.gz wmfod_1_template.nii.gz 0 1

antsRegistrationSyNQuick.sh -d 3 -f wmfod_1_template.nii.gz -m JHU-ICBM-FA-1mm.nii.gz -o JHU_to_wmfod_ -t a

***# register the seed to wmfod***

antsApplyTransforms -d 3 -i seed/cst_r/seed.nii.gz -r wmfod_1_template.nii.gz -o seed/cst_r/seed_a_1mm.nii.gz -t JHU_to_wmfod_0GenericAffine.mat -n NearestNeighbor

antsApplyTransforms -d 3 -i seed/cst_r/exclude.nii.gz -r wmfod_1_template.nii.gz -o seed/cst_r/exclude_a_1mm.nii.gz -t JHU_to_wmfod_0GenericAffine.mat -n NearestNeighbor

antsApplyTransforms -d 3 -i seed/cst_r/target.nii.gz -r wmfod_1_template.nii.gz -o seed/cst_r/target_a_1mm.nii.gz -t JHU_to_wmfod_0GenericAffine.mat -n NearestNeighbor

antsApplyTransforms -d 3 -i seed/cst_l/seed.nii.gz -r wmfod_1_template.nii.gz -o seed/cst_l/seed_a_1mm.nii.gz -t JHU_to_wmfod_0GenericAffine.mat -n NearestNeighbor

antsApplyTransforms -d 3 -i seed/cst_l/exclude.nii.gz -r wmfod_1_template.nii.gz -o seed/cst_l/exclude_a_1mm.nii.gz -t JHU_to_wmfod_0GenericAffine.mat -n NearestNeighbor

antsApplyTransforms -d 3 -i seed/cst_l/target.nii.gz -r wmfod_1_template.nii.gz -o seed/cst_l/target_a_1mm.nii.gz -t JHU_to_wmfod_0GenericAffine.mat -n NearestNeighbor

***#Convert registered seed image to mif***

mkdir cstr

mkdir cstl

mrconvert seed/cst_r/seed_a_1mm.nii.gz cstr/seed.mif

mrconvert seed/cst_r/target_a_1mm.nii.gz cstr/target.mif

mrconvert seed/cst_r/exclude_a_1mm.nii.gz cstr/exclude.mif

mrconvert seed/cst_l/seed_a_1mm.nii.gz cstl/seed.mif

mrconvert seed/cst_l/target.nii.gz cstl/target.mif

mrconvert seed/cst_l/exclude_a_1mm.nii.gz cstl/exclude.mif

***#extract CST***

tckgen -algorithm iFOD2 wmfod_template.mif cstl_2000.tck -seed_image cstl/seed.mif -include cstl/target.mif -exclude cstl/exclude.mif -maxlength 250 -select 2000

tckgen -algorithm iFOD2 wmfod_template.mif cstr_2000.tck -seed_image cstr/seed.mif -include cstr/target.mif -exclude cstr/exclude.mif -maxlength 250 -select 2000

tckedit *.tck cst.tck

***#convert CSTfixel to CSTroi***

tckmap cst.tck -template wmfod_template.mif cst.mif

mrthreshold -abs 3 cst.mif cstroi.mif

***#convert CSTroi to CSTmask***

voxel2fixel cstroi.mif fc fcout cstmask.mif

***# extract the log_FC, FD, and FDC values for the corticospinal tract (CST)***

ls | cut -d "." -f 1 > subj.txt

cat subj.txt

for i in `cat subj.txt`

do

echo $i

#mrdump fd/${i}.mif -mask cstmask.mif fdout/${i}.csv

#mrstats fd/${i}.mif -mask cstmask.mif > fdoutstat/${i}.csv

#cat fdoutstat/${i}.csv >> fdoutstat/fdstat.csv

#mrdump fdc/${i}.mif -mask fdcout/cstmask.mif fdcout/${i}.csv

#mrstats fdc/${i}.mif -mask fdcout/cstmask.mif > fdcoutstat/${i}.csv

#cat fdcoutstat/${i}.csv >> fdcoutstat/fdcstat.csv

#mrdump log_fc/${i}.mif -mask log_fcout/cstmask.mif log_fcout/${i}.csv

#mrstats log_fc/${i}.mif -mask log_fcout/cstmask.mif > log_fcoutstat/${i}.csv

cat log_fcoutstat/${i}.csv >> log_fcoutstat/log_fcstat.csv

done

***#Generate roi for each CST segment***

for i in range(143):

# Create a binary mask

mask = np.zeros_like(data)

mask[:, :, i*part_size:(i+1)*part_size] = 1

part_data = data * mask

part_img = nib.Nifti1Image(part_data, img.affine)

nib.save(part_img, f'part_{i}.nii')

mkdir segmask

ls part_* | cut "." f 1 > segment.txt

nano seg_adjust.sh

for i in `cat segment.txt`

do echo ${i}

voxel2fixel ${i}.nii fd segmask ${i}.mif

done

***#extract value of FBA metrics in each segment of every subject***

mkdir fdoutstatnew

nano segvalue_extract.sh

#!/bin/bash

# Assuming the subj.txt and segment.txt contain the list of subject names and roi names respectively

# Read subjects from subj.txt into an array

subjects=($(cat subj.txt))

# Read roi names from segment.txt into an array

rois=($(cat segment.txt))

# Loop through each subject

for i in "${subjects[@]}"; do

echo "$i" # Echo the current subject

# Loop through each roi for the current subject

for a in "${rois[@]}"; do

# Execute the desired command with the current subject (i) and roi (a)

mrstats "fd/${i}.mif" -mask "segmask/${a}.mif" > "fdoutstatnew/${i}${a}.csv"

done

done
